# Serum Lipid Concentrations Are Associated With Negative Mental Health Outcomes in Healthy Women Aged 35–49 Years

**DOI:** 10.3389/fpsyt.2021.773338

**Published:** 2021-11-02

**Authors:** Jingjie Yu, Zhihui Zhang, Chunjun Li, Jiarui Zhang, Zengbo Ding, Weili Zhu, Qiang Wang

**Affiliations:** ^1^Department of Psychiatry and Psychology, Tianjin Union Medical Center, Tianjin, China; ^2^Stomatology Department, Peking University Third Hospital, Beijing, China; ^3^Health Examination Center, Tianjin Union Medical Center, Tianjin, China; ^4^Beijing Key Laboratory of Drug Dependence, National Institute on Drug Dependence, Peking University, Beijing, China; ^5^Shanghai University of Medicine and Health Sciences Affiliated Zhoupu Hospital, Shanghai, China

**Keywords:** mood disorders, psychosocial stress, obesity, body mass index—BMI, total-cholesterol, LDL-cholesterol, triglyceride, middle-aged women

## Abstract

**Background:** Although the relevant underlying biological mechanisms are still lacking, mental disorders have been closely associated with several metabolic abnormalities including high rates of obesity and metabolic syndrome especially in vulnerable populations. Therefore, the current study aims to examine how metabolic parameters increase the risk for developing mood disorders in individuals stratified by gender and age.

**Methods:** In a routine physical examination, 319 healthy participants were recruited and assigned to six different groups according to age (young adults: 25–34 Y, middle age: 35–49 Y, and older age: 50–65 Y) in both males and females. A linear regression and bivariate correlation analysis were used to analyze the relationship between mood health outcomes measured by the Kessler 10 Psychological Distress Scale (K10) and the metabolic function.

**Results:** The results demonstrated that there was a significant association between K10 scores and metabolic parameters, including Body Mass Index (BMI), total-, LDL-cholesterol, and triglyceride. Furthermore, poor mental health (higher K10 scores) was observed in individuals with increased BMI, total-, LDL-cholesterol, and triglyceride levels particularly in middle-aged women relative to other groups.

**Limitations:** This is a cross-sectional study with a small sample size and lacks longitudinal follow-up evidence and preventive interventions and therefore could not provide the causal inference of metabolic pathophysiology on the increased sensitivity to mental disorders.

**Conclusions:** The potential association suggests that targeting of the metabolic parameters might give us a better understanding of the underlying mechanisms of psychiatric diseases and provide preventive strategies and potential treatment for those with metabolic disturbances especially in middle-aged females.

## Introduction

A growing body of evidence have demonstrated that metabolic syndrome including obesity, changes in cholesterol, triglycerides, lipoproteins, and blood glucose increase the risk of developing mental disorders such as major depressive disorder and anxiety, although the relevant underlying biological mechanisms are still lacking. Psychosocial stress leads to the development and maintenance of both metabolic dysfunctions and psychiatric disorders. There are several biomarkers to illustrate the stressful status such as hair cortisol concentrations are increased in chronically stressed populations due to the dysregulation of the hypothalamic-pituitary-adrenal (HPA) axis (1). Psychological stress also induces chronic neuroendocrine dysregulation leading to metabolic changes that define the metabolic syndrome. Furthermore, chronic stress altered plasma lipids parameters levels including total cholesterol, high-density lipoprotein (HDL) cholesterol, low-density lipoprotein (LDL) cholesterol and triglycerides, and reduced the number of peripheral lymphocytes and subsequently induced immune dysfunction, which is involved in emotional disorders (2). However, evidence showed that not all people with psychiatric disorders display metabolic impairments due to age and gender are the most significant factors influencing the relationship between metabolism and mental disorder. For instance, metabolic conditions (abdominal obesity, high triglyceride, and glucose levels) are associated with an increased risk of a future depressive episode in middle-aged adults (3). Moreover, therapeutic targeting of metabolic parameters might provide new insights into the beneficial effects in depressed patients with high Body Mass Index (BMI) (4). Accordingly, the present study aimed at demonstrating the association between mental health and metabolic measures (i.e., total-, LDL-, and HDL-cholesterol, triglycerides, glucose, and lymphocytes) among heathy young, middle-aged and older individuals and to offer personalized interventions early in the course of the disorder. We hypothesized that abnormal metabolic functions may predict increased mood disorders in vulnerable populations.

## Methods

Data for the current study were collected by a routine physical examination conducted in 319 individuals aged from 25 to 65 years old between January and December 2019. The enrolled subjects were from different job types, such as university staff, doctors in hospital, electrical engineers, office workers, estate agents, and skilled workers. Participants completed a demographic questionnaire including age, gender, relationship status, education, BMI, smoking history, and alcohol consumption. The mental health was assessed by the Chinese version of the Kessler 10 Psychological Distress Scale (K10), which is a 10-item questionnaire developed by Kessler et al. in 2002 and is administered to evaluate distress symptoms in community samples. This scale has brevity, reliability, good precision and strong psychometric properties covering major sociodemographic cases, making it being widely administered in clinical studies as well as in general-purpose health surveys including the annual government health surveys and WHO World Mental Health Surveys (5). The Chinese K10 was administered with a minor modification from the original English version, in which each of the 10 questions relates to an emotional state, and each response has a five-level scale, “1” being “none of the time,” “2” “a little of the time” “3,” “some of the time,” “4” “most of the time,” and “5” “all of the time.” Total scores are ranging from 10 to 50. Hence, a higher score suggests a greater level of psychological distress especially during the past 4 weeks. Considering the sensitivity to determine the risk of samples developing psychiatric disorders, the involvement of K10 assessment in the routine physical examination would fill the gap between community and clinical epidemiology of emotional disorders. Height and weight were measured to calculate BMI [=weight (kg)/height (m)^2^]. The fasting venous blood samples were collected in the morning from all subjects after starved for at least 12 h. Metabolic measures, including total-, HDL-, LDL- cholesterol (mmol/L), triglyceride (mmol/L), fasting glucose (mmol/L), and lymphocytes were measured from the plasma samples of participants using routine standardized laboratorial methods. Considering the various prevalence of stress-related mood disorders in different age populations, we divided the subjects into six groups according to the age and gender: young adults (25–34 Y), middle age (35–49 Y), old age (50–65 Y) in both males and females. The participants with chronic diseases such as diabetes, hypertension, cardiovascular diseases, and other somatic disorders that would affect the metabolism as well as severe nicotine and alcohol abusers were excluded from the data analysis. In addition, the mental health of the subjects was evaluated by an experienced psychiatrist, anyone with psychiatric concern was not included in the data collection. A linear regression model and correlation analysis by Pearson were used to evaluate the possible link between K10 scores and BMI and the lipid concentrations measured. The level of statistical significance was set at *p* < 0.05. Ethical approval was received from the Ethics Committee of Tianjin Union Medical Center (No. 2021B15).

## Results

**Figure 1:**
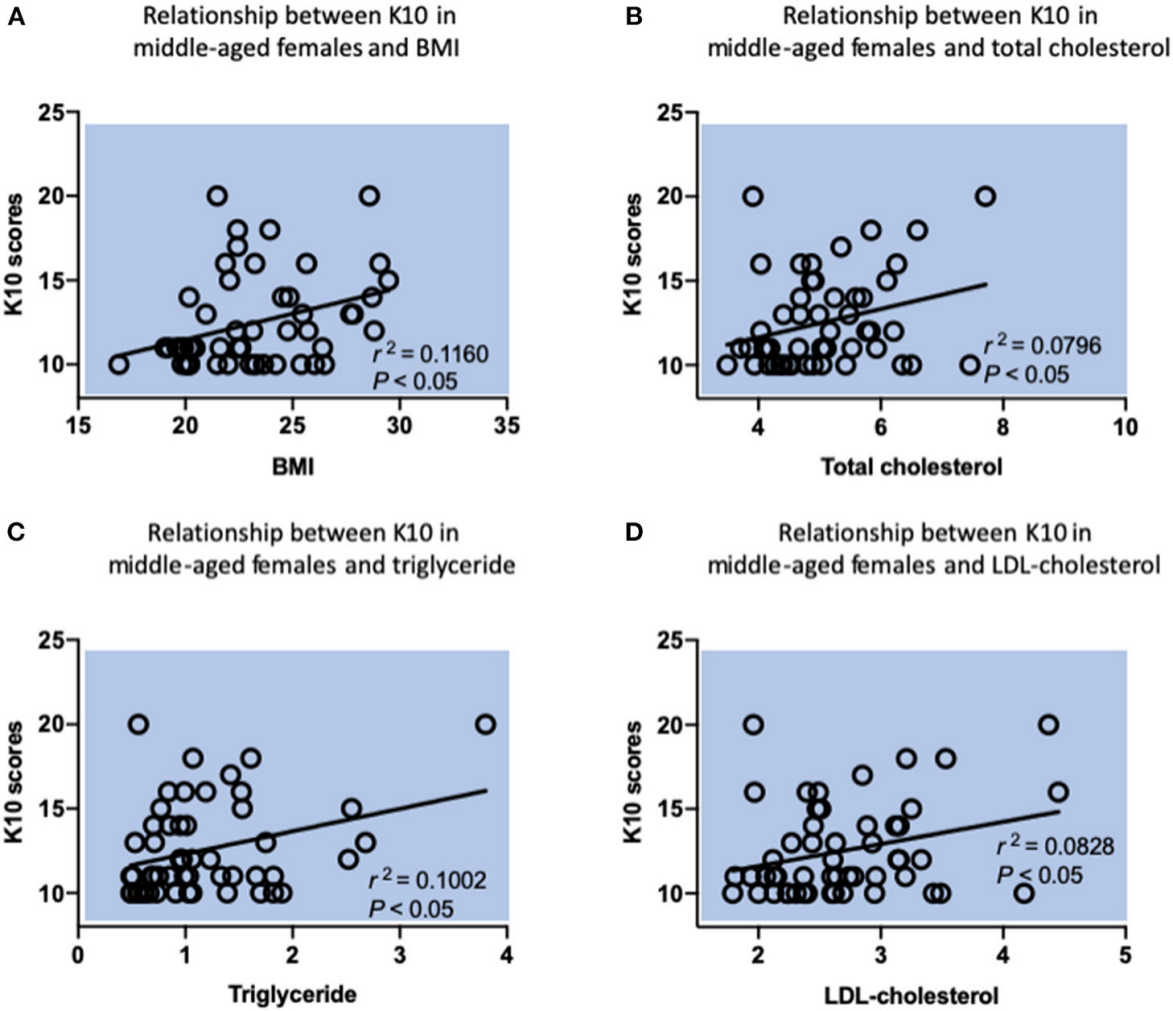
Relationship between K10 scores and metabolic parameters in middle-aged females by Pearson correlation analysis. **(A)** A positive relationship between K10 scores and BMI. *r*^2^ = 0.116, *P* = 0.0135. **(B)** A positive relationship between K10 scores and total cholesterol. *r*^2^ = 0.0796, *P* = 0.0427. **(C)** A positive relationship between K10 scores and triglyceride. *r*^2^ = 0.1002, *P* = 0.0222. **(D)** A positive relationship between K10 scores and LDL-cholesterol. *r*^2^ = 0.0828, *P* = 0.0385. *n* = 52 individuals for this group.

In the whole cohort, the average of K10 scores is 11.69 ± 3.13, suggesting they are in a health psychosocial state. Results of the sociodemographic data, BMI, metabolic characteristics and K10 scores of the sample are summarized in Table 1. The differences in sociodemographic data, physiological and psychological characteristics were assessed by the one-way analysis of variance (ANOVA) and the chi-square test. Bivariate correlation analysis demonstrated that there was a significant association between K10 scores and BMI, total-, LDL-cholesterol, and triglyceride particularly in middle-aged women. We observed that BMI in middle-aged women was positively correlated with psychological distress (*r*^2^: 0.116; 95% CI, 0.075–0.561, *p* = 0.013), followed by an increase of total-cholesterol (*r*^2^: 0.0796; 95% CI, 0.009–0.515, *p* = 0.043), a high level of triglyceride (*r*^2^: 0.1002; 95% CI, 0.048–0.543, *p* = 0.022), and having a high LDL-cholesterol level (*r*^2^: 0.0828; 95% CI, 0.016–0.519, *p* = 0.039) (Figure 1). In female young adults, psychological distress was negatively associated with fasting glucose (*r*^2^: −0.1037; 95% CI,−0.549–0.051 *p* = 0.021). While we did not observe a correlation between fasting glucose and mental health outcome in female middle-aged adults, which is consistent with recent meta-analytic evidence that glucose metabolism was not altered in depressed patients (6). In male older group, the higher scores in K10 and increased total-cholesterol (*r*^2^: 0.1018; 95% CI, 0.0481–0.547, *p* = 0.022), and LDL-cholesterol levels were noted (*r*^2^: 0.0924; 95% CI, 0.031–0.535, *p* = 0.030), showing a significant relationship between total- and LDL-cholesterol and psychological distress. For further information, see Table 2.

**Table 1:** Sociodemographic data, BMI, metabolic characteristics, and K10 scores of the sample.

**Characteristics**	**Total (*N*)**	**Young adults**		**Middle age**		**Old age**		***P***
		**Male**	**Female**	**Male**	**Female**	**Male**	**Female**	
Overall	319	51	51	50	52	51	64	
Age (years)	43.97 ± 12.48	30.78 ± 2.35	29.50 ± 3.06	41.58 ± 4.66	39.75 ± 4.81	58.16 ± 3.89	58.88 ± 4.15	<0.0001
Education (years, Mean ± SD)	16.73 ± 6.01	17.94 ± 4.89	17.87 ± 5.40	17.67 ± 8.05	16.5 ± 7.02	14.42 ± 4.51	11.52 ± 4.31	<0.0001
**Marital status**								
Married	273 (85.6%)	33 (64.7%)	31 (60.8%)	48 (96%)	49 (94.2%)	51 (100%)	61 (95.3%)	<0.0001
Widowed	3 (0.9%)	0	0	1 (2%)	0	0	2 (3.1%)	
Single	43 (13.5%)	18 (35.3%)	20 (39.2%)	1 (2%)	3 (5.8%)	0	1 (1.6%)	
**Working status**								
Employed	272 (85.3%)	51 (100%)	51 (100%)	50 (100%)	51 (98.1%)	40 (78.4%)	29 (45.3%)	<0.0001
Unemployed	0	0	0	0	0	0	0	
Retired	47 (14.7%)	0	0	0	1 (1.9%)	11 (21.6%)	35 (54.7%)	
Smoking	47 (14.7%)	10 (19.6%)	0	12 (24%)	0 (0%)	23 (45.1%)	2 (3.1%)	<0.0001
Drinking	67 (21%)	17 (33.3%)	0	22 (44%)	3 (5.8%)	23 (45.1%)	2 (3.1%)	<0.0001
BMI (Mean ± SD)	24.14 ± 3.33	25.38 ± 3.01	21.01 ± 2.92	25.02 ± 3.18	23.97 ± 3.06	26.46 ± 2.89	23.93 ± 2.23	<0.0001
Total cholesterol (SEM ± SD)	5.15 ± 1.02	4.87 ± 0.73	4.52 ± 0.74	5.08 ± 0.86	4.68 ± 0.93	5.24 ± 0.93	5.90 ± 1.19	<0.0001
Triglyceride (Mean ± SD)	1.46 ± 0.84	1.62 ± 0.82	0.84 ± 0.34	1.94 ± 1.03	1.32 ± 0.66	1.54 ± 0.75	1.61 ± 0.83	<0.0001
HDL-cholesterol (Mean ± SD)	1.44 ± 0.26	1.32 ± 0.25	1.55 ± 0.23	1.32 ± 0.20	1.52 ± 0.24	1.31 ± 0.23	1.54 ± 0.27	<0.0001
LDL-cholesterol (Mean ± SD)	2.74 ± 0.63	2.57 ± 0.45	2.34 ± 0.46	2.73 ± 0.59	2.41 ± 0.61	2.82 ± 0.57	3.18 ± 0.71	<0.0001
Fasting glucose (Mean ± SD)	5.54 ± 1.45	5.19 ± 0.44	4.96 ± 0.29	5.52 ± 1.02	5.16 ± 1.62	6.44 ± 2.54	5.79 ± 1.06	<0.0001
Lymphocytes (Mean ± SD)	0.35 ± 0.077	0.36 ± 0.075	0.36 ± 0.076	0.36 ± 0.070	0.37 ± 0.083	0.33 ± 0.063	0.36 ± 0.080	0.1648
K10 scores (Mean ± SD)	11.71 ± 3.13	12.29 ± 2.99	12.20 ± 3.60	11.42 ± 2.29	14.75 ± 2.78	10.31 ± 0.84	11.52 ± 4.31	0.0033

**Table 2:** Multivariate general linear regression, stratified by age and sex.

**Mood outcome**	**Biological**	**Whole cohort**	**Yong adults**		**Middle age**		**Old age**	
	**measures**	**(*N* = 319)**	**(25–34 Y)**		**(35–49 Y)**		**(50–65 Y)**	
			**M (*N* = 51)**	**F (*N* = 51)**	**M (*N* = 50)**	**F (*N* = 52)**	**M (*N* = 51)**	**F (*N* = 64)**
K10 scores	BMI	(0.175) [−0.184, 0.034]	(0.573) [−0.369, 0.207]	(0.105) [−0.413, 0.040]	(0.927) [−0.420, 0.384]	(**0.013**) [0.075, 0.561]	(0.366) [−1.43, 0.537]	(0.955) [−0.135, 0.127]
	Total cholesterol	(0.115) [−0.064, 0.007]	(0.378) [−0.100, 0.039]	(0.436) [−0.082, 0.036]	(0.214) [−0.174, 0.040]	(**0.043**) [0.003 to 0.185]	(**0.022**) [0.053, 0.656]	(0.219) [−0.112, 0.026]
	Triglyceride	(0.505) [−0.039, 0.019]	(0.566) [−0.056, 0.101]	(0.439) [−0.037, 0.016]	(0.276) [−0.199, 0.058]	(**0.022**) [0.011, 0.139]	(0.069) [−0.019, 0.476]	(0.579) [−0.062, 0.035]
	HDL-cholesterol	(0.712) [−0.008, 0.011]	(0.379) [−0.034, 0.013]	(0.794) [−0.021, 0.016]	(0.841) [−0.028, 0.023]	(0.99) [−0.025, 0.025]	(0.452) [−0.048, 0.106]	(0.749) [−0.018, 0.013]
	LDL-cholesterol	(0.141) [−0.039, 0.0056]	(0.505) [−0.057, 0.028]	(0.505) [−0.048, 0.024]	(0.261) [−0.116, 0.032]	(**0.039**) [0.016, 0.519]	(**0.030**) [0.021, 0.391]	(0.197) [−0.068, 0.014]
	Fasting glucose	(0.259) [−0.080, 0.022]	(0.422) [−0.059, 0.025]	(**0.021**) [−0.048, −0.004]	(0.912) [−0.122, 0.136]	(0.287) [−0.077, 0.253]	(0.845) [−0.956, 0.786]	(0.932) [−0.059, 0.065]
	Lymphocytes	(0.537) [−0.002, 0.004]	(0.367) [−0.004, 0.010]	(0.681) [−0.007, 0.005]	(0.171) [−0.003, 0.015]	(0.964) [−0.009, 0.008]	(0.137) [−0.005, 0.037]	(0.779) [−0.006, 0.004]
	Marital status	(0.018) [−0.027, −0.0026]	(0.720) [−0.038, 0.055]	(0.870) [−0.036, 0.043]	(0.568) [−0.032, 0.0178]	(0.580) [−0.0307, 0.0174]		(0.729) [−0.015, 0.010]
	Education	(0.246) [−0.127, 0.033]	(0.922) [−0.059, 0.054]	(0.589) [−0.047, 0.082]	(0.167) [−0.073, 0.412]	(0.738) [−0.117, 0.164]	(0.799) [−1.202, 0.930]	(0.421) [−0.076, 0.179]
	Smoking	(0.829) [−0.061, 0.049]	(0.320) [−0.522, 0.174]		(0.195) [−0.089, 0.0186]		(0.943) [−0.179, 0.166]	(0.618) [−0.013, 0.008]
	Drinking	(0.490) [−0.020, 0.009]	(0.104) [−0.009, 0.096]		(0.341) [−0.033, 0.093]	(0.474) [−0.015, 0.033]	(0.877) [−0.159, 0.186]	(0.618) [−0.013, 0.008]

*The multivariate general linear model in lipid concentrations and mood outcomes; stratified by age groups (25–34, 35–49, 50–65 years old) and sex. Expressed by p-values are in parentheses and 95% confidence intervals are in square brackets. The bold values mean significant results. M, male; F, female; K10, Kessler 10 Psychological Distress Scale*.

## Discussion

In current study, we investigated the impact of metabolic functions on the associations of mental health outcomes by assessment of psychosocial stress in healthy subjects. To our knowledge, our study is the first one to explore the potential relationship between metabolic measures and mental state in health subjects stratified by age and gender. Our results demonstrated that poor mental health was significant associated with increased BMI, total-, LDL-cholesterol, and triglyceride levels in healthy middle-aged women but not in young or older adults. These findings support the notion that women are more vulnerable to mental health disorders than men, and specifically, middle-aged women are likely to have an increased risk of obesity and poor mental health due to reaching the menopause, changing marital or socioeconomic status, and unhealthy life-style habits (7). The negative and reciprocal impact of metabolic syndrome (i.e., triglycerides, fasting glucose, and obesity) in middle-aged females on the physical, psychological, emotional, and behavioral responses toward a stressful event has been evidenced (8), emphasizing the notion that susceptibility to stress exposure and metabolic dysregulations and their consequences are responsible for the elevated risk of psychiatric disorders. Particularly, female participants experiencing high stress showed higher low-density lipoprotein levels compared to the low stress group (9), suggesting that a higher level of blood lipids and lipoprotein is correlated with psychological stress associated with high prevalence of emotional disorders.

The bidirectional associations between mental health and metabolic disturbances have been intensively evidenced although it still remains unclear whether the risk factors are cause or consequence. Nevertheless, the association between higher BMI and greater serum cholesterols levels and increased risk of mental disorders, e.g., being overweight is positively associated with increased risk to depression has been confirmed by several community-based cross-sectional studies particularly in women but not in men (10, 11). Since the detailed insight into the biological mechanisms linking depression and metabolic impairment is not fully clarified, the longitudinal studies are indispensable in producing more evidence on the bidirectional association.

Given the numerous studies reporting increased lipid metabolism levels involved in psychiatric disease, the association between low levels of serum cholesterol and mental illnesses, such as depression and other stress-related mental illnesses, could not be dismissed. For example, it has been proposed that cholesterol levels were reduced in patients with major depressive disorder possibly *via* disruption the availability of serotonergic receptors, which are major targets implicated in depression pathophysiology and in the mechanism of antidepressant action (12, 13). Further evidence supporting the complex relationship between lipid metabolism and mood disorders and the exact regulation is required.

Our data did not show an association between the immune marker lymphocytes and the mental health among different age groups, indicating that peripheral immune system is not a central process to induce the development of mood and metabolic alterations in the current cohort. Notably, neutrophil to lymphocyte ratio, but not absolute lymphocytes counts, was used to explore the biological mechanisms underlying psychiatric disorders (14, 15). The specific relationship between blood immune markers and mental status needs further examination. According to the current data, the metabolic parameters (total and LDL-cholesterol) also correlated with the K10 scores in male older participants. There may be multiple factors rather than metabolic signal contributing to this association in this subgroup. It is worth noting that the psychosocial stress from retirement and subsequent alterations in social connection, economic status, and environmental opportunities may increase the negative consequences of mental health in male old adults (16). However, women also exhibit better psychological resilience than men following retirement (17). So we did not observe this association between retirement and the mental health in older female group.

Given the evidence of altered lipid metabolism in vulnerable populations, lifestyle factors such as diet, exercise, and physical complications influencing metabolic process have also been ascertained in the development, progression and treatment of mental health disorders. For example, depressive symptoms are also positively associated with the high consumption of fast food (18), low levels of physical activity (especially in women and those aged 40 years and older) (19), and reduced sleep quality (20) through regulation of several physiological pathways involved in mood disorders. Therefore, future mental health interventions targeting these lifestyle factors would enhance the outcome of interventions associated with psychosocial stress symptoms and metabolic dysfunction.

Concerning the limitations, the current study shows the potential correlation based on observational data from routine physical examination and inevitably results in an underestimate or overestimate of the causal inference due to confounding, selection and measurement biases. Applications of statistical and design-based methods are required to minimize potential bias and establish an improved estimation of the causal inference. In addition, this is a cross-sectional study with a small sample size and lacks longitudinal follow-up evidence and preventive interventions and therefore could not provide the causal inference of metabolic pathophysiology on the increased sensitivity to mental disorders. Future studies aiming at determining the relationship between metabolic indicators and mood outcomes in a large sample size with intervention and follow-up design are needed to provide an early prediction and treatment in vulnerable individuals such as middle-aged women. Moreover, the fact that the high education level of the recruited participants (average is more than 16 years) lack of the representative of all respondents with psychological distress makes it difficult to extend the current findings to general population and reduces the generalizability of our results.

In conclusion, we found that metabolic risk factors affect psychosocial stress in middle-aged female adults and reciprocally changing the coping style to psychological distress may reduce the development of the metabolic syndrome in women. Our findings reveal that metabolic conditions may play an important role in predicting outcomes for middle-age female patients with high risk for mood disorders, raising the possibility that metabolic functions should be seriously taken into consideration not only for improving psychosocial stress response such as health lifestyle, diminished stress, physical activity, and weight-loss interventions in an early stage but also for providing precise interventions for mood disorders in vulnerable middle-aged women.

## Data Availability Statement

The original contributions presented in the study are included in the article/supplementary material, further inquiries can be directed to the corresponding author/s.

## Ethics Statement

The studies involving human participants were reviewed and approved by Ethics Committee of Tianjin Union Medical Center (No. 2021B15). The patients/participants provided their written informed consent to participate in this study.

## Author Contributions

JY, WZ, CL, JZ, and ZD initiated the study. JY obtained ethics approval. CL, JZ, ZZ, and ZD analyzed the data. JY, ZZ, and WZ wrote a first draft of the study protocol. WZ, ZZ, and QW obtained funding for the study and critically revised the manuscript. WZ and QW supervised the project and provided substantial contributions to the paper. All authors participated in the conception of the study and approved the final manuscript.

## Funding

This work was supported by the National Natural Science Foundation of China (No. 81800983), Key Basic Research Project of Shandong Provincial Natural Science Foundation (ZR2019ZD27), Peking University Medicine Seed Fund for Interdisciplinary Research (Nos. BMU2020MX022 and 71006Y2337), Key Clinical Projects of Peking University Third Hospital (No. BYSYZD2019035), and State Key Laboratory of Environmental Chemistry and Ecotoxicology, Research Center for Eco-Environmental Sciences, Chinese Academy of Sciences (KF2020-18).

## Conflict of Interest

The authors declare that the research was conducted in the absence of any commercial or financial relationships that could be construed as a potential conflict of interest.

## Publisher's Note

All claims expressed in this article are solely those of the authors and do not necessarily represent those of their affiliated organizations, or those of the publisher, the editors and the reviewers. Any product that may be evaluated in this article, or claim that may be made by its manufacturer, is not guaranteed or endorsed by the publisher.
